# Molecular Polarizability under Vibrational Strong Coupling

**DOI:** 10.1021/acs.jctc.5c00461

**Published:** 2025-05-14

**Authors:** Thomas Schnappinger, Markus Kowalewski

**Affiliations:** Department of Physics, 132068 Stockholm University, AlbaNova University Center, SE-106 91 Stockholm, Sweden

## Abstract

Polaritonic chemistry offers the possibility of modifying molecular properties and even influencing chemical reactivity through strong coupling between vibrational transitions and confined light modes in optical cavities. Despite considerable theoretical progress, and due to the complexity of the coupled light-matter system, the fundamental mechanism of how and if collective strong coupling can induce local changes in individual molecules is still unclear. We derive an analytical formulation of static polarizabilities within linear-response theory for molecules under strong coupling using the cavity Born–Oppenheimer Hartree–Fock ansatz. This ab-initio method consistently describes vibrational strong coupling and electron–photon interactions even for ensembles of molecules. For different types of molecular ensembles, we observed local changes in the polarizabilities and dipole moments that are induced by collective strong coupling. Furthermore, we used the polarizabilities to calculate vibro-polaritonic Raman spectra in the harmonic approximation. This allows us to comprehensively compare the effect of vibrational strong coupling on IR and Raman spectra on an equal footing.

## Graphical abstract

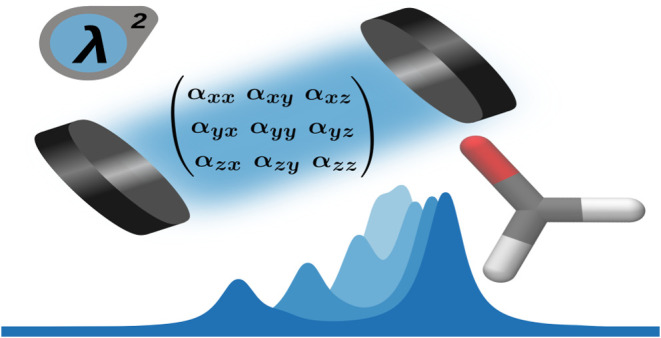

## 1 Introduction

By placing molecules in a modified photonic environment, such as a Fabry–Pérot cavity, we can form hybrid states known as polaritons. These polaritons are hybrid light–matter states that emerge from the resonant exchange of energy between an optically bright transition in matter and the confined photonic mode of the optical cavity.^1–4^ The spectroscopic signature of such a strong light-matter coupling is the formation of a lower polariton (LP) transition and an upper polariton (UP) transition separated by the Rabi splitting frequency, Ω_*R*_, which results from a distinct splitting of the peak in the absorption spectrum.^5,6^ A particularly fascinating phenomenon occurs when the light field is strongly coupled to molecular vibrations, called vibrational strong coupling (VSC), where it seems possible to influence ground state reactions^7–12^ in the “dark”, i.e., without external illumination. Despite considerable experimental and theoretical efforts in recent years,^13–25^ a fundamental understanding of the underlying microscopic and macroscopic physical mechanisms of VSC is still lacking. An atomistic understanding of the phenomenon is challenging due to the complexity of its collective nature. However, a self-consistent treatment of the field-dressed electronic structure in a molecular ensemble appears to be a crucial ingredient in the description of cavity-induced microscopic changes under collective strong coupling.^18,20,26–30^ The cavity Born–Oppenheimer Hartree–Fock (CBO-HF) approach^18^ is a formulation of the well-known Hartree–Fock ansatz in the cavity Born–Oppenheimer approximation (CBOA)^31–33^ that treats the dressed electron–photon system in an ab-initio fashion. Using the CBO-HF framework, we have identified local strong coupling effects in molecular ensembles^18,20^ and simulated vibro-polaritonic IR spectra within the harmonic approximation.^28^

The molecular polarizability ***α*** is an important property that describes how the electronic structure reacts under the influence of an external electric field. For example, the change in polarizability determines whether a vibrational mode is Raman active or not.^34^ In addition to defining light-matter interactions, molecular polarizabilities also influence how molecules interact by defining dispersion interactions,^35,36^ which are, for example, essential for understanding solvation processes. The maxima of the static polarizability along a bond dissociation coordinate can be used to define cut-off points for bond cleavage,^37^ as they represent the beginning of the localization of the shared electron density in the constituent fragments. Furthermore, polarizabilities are relevant in the context of strong light-matter coupling. It has been reported^11^ that in experiments, VSC can modify the London dispersion forces, or, in other words, the underlying molecular polarizability under collective strong coupling. Using static polarizabilities, it is possible to estimate the influence of cavity interactions on molecular geometries.^38^

Within the framework of the CBO-HF ansatz, we derive an analytic expression for the static polarizability ***α*** as the second derivative of the energy with respect to an external electric field using linear-response theory. To validate the linear-response approach, we compare ***α*** with numerically computed values by explicitly including an external electric field in the electronic CBOA Hamiltonian and solving the CBO-HF equation. We then investigate how polarizabilities and permanent dipole moments change under the VSC in the case of a single molecular system and small molecular ensembles for a set of identical molecules. In the first part, we investigate whether cavity-mediated collective interactions modify the dipole moments and polarizabilities of individual molecules in an ensemble. This type of local modification of ***α*** under VSC has recently been shown for a full-harmonic model.^39^ For this full-harmonic model, each molecule in an ensemble coupled to a single cavity mode is described by including all nuclei and a single effective electron bound by a harmonic interactions. In the second part of the manuscript, we use the analytical polarizability to extend our recent work on ab-initio vibro-polaritonic spectra^28^ to determine Raman spectra for molecules under VSC. Raman scattering is a useful method for obtaining information about material properties and chemical structures, especially when probing the rovibrational structure. However, the influence of VSC on Raman scattering is still under discussion on both the experimental^40–42^ and theoretical^43–46^ sides. Here, we investigate the different effects of VSC on IR and Raman spectra for a single formaldehyde molecule coupled to two cavity modes in a simplified Fabry–Pérot-like setup.

## 2 Theory

The starting point for ab-initio studies of VSC is the CBOA^31–33^ in the length gauge and dipole approximation. Within CBOA, the cavity field modes are grouped with the nuclei in a generalized Born–Huang expansion,^47,48^ which allows one to first solve the quantum problem of the electrons for a fixed nuclear and photonic configuration. The combined nuclear-photonic problem can subsequently be solved fully quantum mechanically or semi-classically. The electronic CBOA Hamiltonian for *N*_*M*_ cavity field modes after the adiabatic separation reads

1 $${Ĥ}_{\mathrm{CBO}}={Ĥ}_{\mathrm{el}}+\sum_{c=1}^{N_{M}}\frac{\omega_{c}^{2}}{2}{\left({\mathrm{q̂}}_{c}-\frac{{\mathrm{d̂}}_{c}}{\omega_{c}}\right)}^{2}$$

where *Ĥ*_*el*_ is the Hamiltonian for the field-free many-electron system, *q*_*c*_ is the photon displacement coordinate, ω_*c*_ is the frequency of the cavity mode *c*, and *N*_*M*_ is the number of modes. The linear coupling between the molecular system and the photon displacement field, as well as the dipole self-energy (DSE) contribution,^49,50^ which describes the self-polarization of the molecule-cavity system, is characterized by the dressed dipole operator *d̂*_*c*_. This operator is defined as the scalar product of the standard dipole operator ***μ* ^** and the coupling parameter ***λ***_*c*_

2 $${\mathrm{d̂}}_{c}=\lambda_{c}\cdot \mathrm{\mu ̂}=\lambda_{c}\cdot \left({\mathrm{\mu ̂}}_{\mathrm{el}}+\mu_{\mathrm{nuc}}\right)\;\mathrm{with}\;\lambda_{c}=e_{c}\lambda_{c}=e_{c}\sqrt{\frac{4\pi }{V_{c}}}$$

The unit vector ***e***_*c*_ denotes the polarization axis of the cavity mode, and *λ*_*c*_ is defined by the effective mode volume $V_{c}$ of the corresponding cavity mode. For simplicity, we assume that $V_{c}$ corresponds to the physical volume *V*. To go beyond the case of a single molecule, the collective coupling strength ***λ***_*c*_ is kept constant by applying a scaling factor of $1/\sqrt{N_{\mathrm{mol}}}$ to obtain a fixed Rabi splitting for different ensemble sizes

3 $$\lambda_{c}=e_{c}\frac{\lambda_{0}}{\sqrt{N_{\mathrm{mol}}}}$$

Here, *λ*_0_ is treated as a tunable coupling parameter and is equivalent to *λ*_*c*_ in eq 2 for a single molecule. As a result of the rescaling, we increase the physical volume *V* of the cavity, but by including more molecules, we effectively keep the average density of molecules *N*_*mol*_/*V* fixed.

In our recent work,^18,28^ we have introduced the CBO-HF approach, which represents a formulation of the well-known Hartree–Fock ansatz in the context of the CBOA. As in standard Hartree–Fock, the many-electron wave function is a single Slater determinant *Ψ*, which is optimized for a fixed nuclear configuration and a set of photon displacement coordinates. In the following, we reformulate the optimization in terms of an exponential parametrization

4 $$\left|\Psi \left(\kappa \right)\right\rangle =e^{\mathrm{\kappa ̂}}\left|\Psi_{0}\right\rangle$$

where *e* ^*κ̂*^ is a unitary operator that performs rotations between occupied and virtual spin orbitals in a reference determinant wave function *Ψ*_0_. The single-excitation operator *κ̂* in the second-quantization formalism can be written as

5 $$\mathrm{\kappa ̂}=\sum_{a}^{\mathrm{occ}}\sum_{r}^{\mathrm{virt}}\left(\kappa_{\mathrm{ar}}{â}_{a}^{†}{â}_{r}-\kappa_{\mathrm{ar}}{â}_{r}^{†}{â}_{a}\right)$$

where *â*_*k*_^†^ and *â*_*k*_ are the Fermionic creation and annihilation operators of the spin orbital *k*, respectively, and *κ*_*ar*_ are the orbital rotation parameters. In each optimization step, the orbitals in the reference determinant *Ψ*_0_ are updated so that variations of the orbital rotation parameters around ***κ*** = 0 are always considered. The converged CBO-HF energy then reads:

6 $$E_{\mathrm{CBO}}\left(\kappa \right)=\left\langle \Psi \left(\kappa \right)\left|{Ĥ}_{\mathrm{CBO}}\right|\Psi \left(\kappa \right)\right\rangle$$

Using the converged Slater determinant *Ψ*(***κ***), the expectation values of the dipole moment^51^ can be calculated with the corresponding dipole operator

7 $$\left\langle \mathrm{\mu ̂}\rangle_{\mathrm{CBO}}=\left\langle \Psi \left(\kappa \right)\left|{\mathrm{\mu ̂}}_{\mathrm{el}}\right|\Psi \left(\kappa \right)\right\rangle +\mu_{\mathrm{nuc}}=-\sum_{a}^{\mathrm{occ}}\left\langle a\right|\mathrm{r̂}\right|a\rangle +\sum_{A}^{N_{\mathrm{nuc}}}Z_{A}R_{A}$$

where *a* are the occupied spin orbitals, ***r* ^** and ***R***_*A*_ are the electronic and nuclear position vectors, and *Z*_*A*_ is the nuclear charge.

### 2.1 CBO-HF Polarizability

The definition of the static polarizability tensor ***α*** of a molecular system is the response of its dipole moment ⟨ ***μ* ^**⟩ to a static external electric field ***F***, which is equivalent to the second derivative of the electronic energy *E* with respect to ***F*** ^52^

8 $$\left\langle \alpha_{\mathrm{ij}}\right\rangle =\frac{\partial \left\langle {\mathrm{\mu ̂}}_{i}\right\rangle }{\partial F_{j}}=\frac{\partial^{2}E}{\partial F_{i}\partial F_{j}}$$

This polarizability can be calculated self-consistently by explicitly including ***F*** in the electronic CBOA Hamiltonian

9 $${Ĥ}_{\mathrm{CBO}}^{F}={Ĥ}_{\mathrm{el}}+\sum_{c=1}^{N_{M}}\frac{\omega_{c}^{2}}{2}{\left({\mathrm{q̂}}_{c}-\frac{{\mathrm{d̂}}_{c}}{\omega_{c}}\right)}^{2}-\left({\mathrm{\mu ̂}}_{\mathrm{el}}+\mu_{\mathrm{nuc}}\right)F$$

The converged CBO-HF energy then reads

10 $$E_{\mathrm{CBO}}^{F}\left(\kappa \right)=\left\langle \Phi \left(\kappa \right)\left|{Ĥ}_{\mathrm{CBO}}^{F}\right|\Phi \left(\kappa \right)\right\rangle$$

The converged Slater determinant *Φ*(***κ***), explicitly taking into account ***F***, is then used to determine ⟨ ***μ* ^**⟩_CBO_^***F***^ = ⟨*Φ* (***κ***)| ***μ* ^**|*Φ* (***κ***)⟩. The CBO-HF polarizability ⟨ ***α*** ⟩_CBO_^num^ can be approximated numerically via finite differences

11 $$\langle \alpha_{\mathrm{ij}}\rangle_{\mathrm{CBO}}^{\mathrm{num}}\approx \frac{\langle {\mathrm{\mu ̂}}_{i}\rangle_{\mathrm{CBO}}^{F_{j}}-\langle {\mathrm{\mu ̂}}_{i}\rangle_{\mathrm{CBO}}^{-F_{j}}}{2F_{j}}$$

It should be noted that this formula has a leading error term that is quadratic in the applied field and depending on the used field strength the results can be contaminated by higher order responses (hyperpolarizabilities).^53^

Alternatively, the polarizability ⟨ ***α*** ⟩_CBO_^cphf^ can be calculated using linear-response theory^54^

12 $$\langle \alpha_{\mathrm{ij}}\rangle_{\mathrm{CBO}}^{\mathrm{cphf}}=-\left[\frac{\partial^{2}E_{\mathrm{CBO}}^{0}}{\partial F_{i}\partial F_{j}}+\sum_{a}^{\mathrm{occ}}\sum_{r}^{\mathrm{virt}}{\frac{\partial^{2}E_{\mathrm{CBO}}^{0}}{\partial F_{i}\partial \kappa_{\mathrm{ar}}}|}_{\kappa =0}\frac{\partial \kappa_{\mathrm{ar}}}{\partial F_{j}}\right]$$

Here, *E*_CBO_^0^ = ⟨*Ψ* (***κ***)|*Ĥ*_CBO_^***F***^|*Ψ* (***κ***)⟩ is the CBO-HF expectation value of *Ĥ*_CBO_^***F***^ calculated using the Slater determinant *Ψ* (***κ***) optimized without the external field. Since only the first derivative of *Ĥ*_CBO_^**F**^ (see eq 9) with respect to the field is nonzero, i.e., the Hellman–Feynman term, *E*_CBO_^0^ depends only linearly on the external field. Consequently, its second derivative with respect to ***F***, the first term in eq 12, is zero. The second term interpretable as a perturbed electronic gradient is

13 $${\frac{\partial^{2}E_{\mathrm{CBO}}^{0}}{\partial F_{i}\partial \kappa_{\mathrm{ar}}}|}_{\kappa =0}=-2\frac{\partial \left\langle \Psi_{a}^{r}\left|{Ĥ}_{\mathrm{CBO}}^{F}\right|\Psi \left(\kappa \right)\right\rangle }{\partial F_{i}}=-2\left\langle r\left|{\mathrm{\mu ̂}}_{\mathrm{el}}^{i}\right|a\right\rangle$$

where *a* and *r* are an occupied spin orbital and a virtual spin orbital, respectively, *Ψ* (***κ***) is the CBO-HF Slater determinant optimized without the external field, and *Ψ*_*a*_^*r*^ is the corresponding single-excitation determinant. Due to Brillouin’s theorem, all contributions of *Ĥ*_CBO_^ext^, except the dipole interaction with the external field, are zero. The only remaining part is the wave-function linear-response vector ∂*κ*_*ar*_/*F*_*j*_, which requires solving the linear-response equations that are the CBO-HF counterparts of the coupled-perturbed Hartree–Fock (CPHF) equations in the cavity-free case. These CBO-HF linear-response equations read

14 $$\sum_{b}^{\mathrm{occ}}\sum_{s}^{\mathrm{virt}}\left(A_{\mathrm{ar},\mathrm{bs}}+B_{\mathrm{ar},\mathrm{bs}}\right)\frac{\partial \kappa_{\mathrm{ar}}}{\partial F_{j}}=\left\langle r\left|{\mathrm{\mu ̂}}_{\mathrm{el}}^{j}\right|a\right\rangle$$

Working within the framework of the CBO-HF ansatz, the two terms *A*_*ar*,*bs*_ and *B*_*ar*,*bs*_ contain the standard electron repulsion integrals and the corresponding two-electron DSE contributions

15 $$\begin{array}{ll} A_{\mathrm{ar},\mathrm{bs}}=\left(\epsilon_{r}-\epsilon_{a}\right)\delta_{\mathrm{rs}}\delta_{\mathrm{ab}}+\left\langle \mathrm{rb}|\mathrm{as}\right\rangle -\left\langle \mathrm{rb}|\mathrm{sa}\right\rangle & \left(15\right)\\ +\sum_{c}^{N_{M}}\left\langle r\left|{\mathrm{d̂}}_{c}\right|a\right\rangle \left\langle b\left|{\mathrm{d̂}}_{c}\right|s\right\rangle -\left\langle r\left|{\mathrm{d̂}}_{c}\right|s\right\rangle \left\langle b\left|{\mathrm{d̂}}_{c}\right|a\right\rangle & \left(16\right) \end{array}$$

and

17 $$\begin{array}{ll} B_{\mathrm{ar},\mathrm{bs}}=\left\langle \mathrm{rs}|\mathrm{ab}\right\rangle -\left\langle \mathrm{rs}|\mathrm{ba}\right\rangle & \left(17\right)\\ +\sum_{c}^{N_{M}}\left\langle r\left|{\mathrm{d̂}}_{c}\right|s\right\rangle \left\langle a\left|{\mathrm{d̂}}_{c}\right|b\right\rangle -\left\langle r\left|{\mathrm{d̂}}_{c}\right|b\right\rangle \left\langle s\left|{\mathrm{d̂}}_{c}\right|a\right\rangle . & \left(18\right) \end{array}$$

Here, *ϵ*_*k*_ is the orbital energy of the spin orbital *k*. Using vector/matrix notations, the CPHF polarizability ⟨ ***α*** ⟩_CBO_^cphf^ can be written as

19 $$\langle \alpha_{\mathrm{ij}}\rangle_{\mathrm{CBO}}^{\mathrm{cphf}}=2\mu_{i}^{T}{\left(A+B\right)}^{-1}\mu_{j}$$

where ***μ***_*i*_ is the vector of components *μ*_*i*,*ar*_ = ⟨*r*|*μ̂*_*el*_^*i*^| *a*⟩ and (***A*** + ***B***) is the matrix of elements (*A*_*ar*,*bs*_ + *B*_*ar*,*bs*_). In this work, we consider two variations of ⟨ ***α*** ⟩_CBO_^cphf^, in one case, the DSE two-electron integrals (eqs 16 and 18) are included, and in the second case, only the standard electron repulsion integrals are considered (eqs 15 and 16). Note that in both cases, all necessary quantities are obtained from a single CBO-HF calculation.

### 2.2 CBO-HF Raman Activity in the Harmonic Approximation

To determine Raman activities, i.e., scattering factors, for a molecular system under VSC, we extend our recently presented approach for vibro-polaritonic spectra in the harmonic approximation.^28^ The derivative of the polarizability ∇_*k*_ ***α*** with respect to the *k*th vibro-polaritonic normal mode vector ***Q*** ^*k*^ reads

20 $$\nabla_{k}\alpha_{\mathrm{ij}}=\sum_{n=1}^{N_{A}+N_{M}}{\left(\nabla^{\mathrm{ij}}\langle \alpha \rangle_{\mathrm{CBO}}\right)}_{n}\cdot \frac{Q_{n}^{k}}{\sqrt{M_{n}}}$$

where the summation runs over the Cartesian coordinates *N*_*A*_ and photo-displacement coordinates *N*_*M*_. The normal mode vector is rescaled by the square root of the corresponding atomic masses, and a mass of one is used for the photonic components. The necessary polarizability gradient ***∇*** ⟨ ***α*** ⟩_CBO_ is calculated using finite differences based on the polarizabilities determined numerically or by the linear response formalism, both described in Section 2.1. The elements ∇_*k*_ ***α***_*ij*_ are used to determine the mean polarizability^34,55^ *α̅*_*k*_ for the vibro-polaritonic normal mode *k*

21 $${\mathrm{\alpha ̅}}_{k}=\left(\nabla_{k}\alpha_{\mathrm{xx}}+\nabla_{k}\alpha_{\mathrm{yy}}+\nabla_{k}\alpha_{\mathrm{zz}}\right)/3$$

as well as the anisotropy *γ*_*k*_ ^34,55^

22 $$\gamma_{k}^{2}={\left(\nabla_{k}\alpha_{\mathrm{xx}}-\nabla_{k}\alpha_{\mathrm{yy}}\right)}^{2}/2+{\left(\nabla_{k}\alpha_{\mathrm{yy}}-\nabla_{k}\alpha_{\mathrm{zz}}\right)}^{2}/2+{\left(\nabla_{k}\alpha_{\mathrm{zz}}-\nabla_{k}\alpha_{\mathrm{xx}}\right)}^{2}/2+3\left({\left(\nabla_{k}\alpha_{\mathrm{xy}}\right)}^{2}+{\left(\nabla_{k}\alpha_{\mathrm{yz}}\right)}^{2}+{\left(\nabla_{k}\alpha_{\mathrm{xz}}\right)}^{2}\right)$$

Combining both mean polarizability *α̅*_*k*_ and anisotropy *γ*_*k*_ yields the corresponding Raman activity. The scattering factor *S*_*k*_ for the vibro-polaritonic normal mode *k* is calculated as follows

23 $$S_{k}=45{\mathrm{\alpha ̅}}_{k}^{2}+7\gamma_{k}^{2}$$

## 3 Computational Details

The calculation of the numerical and CPHF static polarizability with the CBO-HF ansatz, as well as the calculation of Raman activities, has been implemented in the Psi4NumPy environment,^56^ which is an extension of the PSI4 ^57^ electronic structure package. All molecular structures used are based on geometries of a single molecule optimized outside the cavity at the Hartree–Fock level of theory using the aug-cc-pVDZ basis set.^58^ To study the collective effects of VSC on the static polarizability and the permanent dipole moment, we used the optimized geometries to create small ensembles by placing *N*_*mol*_ replicas of the single molecule separated by 100 Å inside the cavity. This intermolecular separation is chosen to avoid non-cavity-induced interactions between the molecules. The individual molecular dipole moments and, in the case of CO_2,_ the molecular axes are aligned in parallel to each other. These ensembles of *N*_*mol*_ molecules are placed at the maximum of the cavity field and are oriented parallel to the polarization axis of a single cavity mode. To demonstrate the influence of VSC on Raman activities, a single formaldehyde molecule interacting with two orthogonal cavity modes is studied. One cavity mode is aligned with the carbonyl group, and the second orthogonal mode is in the molecular plane. As shown in the literature,^38,46,59^ the effects on the internal coordinates are small for the coupling strengths studied here; consequently, we do not re-optimize the geometries of the molecular systems in the cavity. Molecular reorientation plays an important role, especially in the case of a single cavity mode. For linear molecules, the most energetically favorable orientations are those in which the molecules are not coupled to the cavity field at all, that is, the cavity polarization axis is orthogonal to the molecular dipole moment.^18,38^ However, depending on the coupling strength, the corresponding rotational barriers can be quite small, and to define an upper bound for strong coupling effects, we orient the molecules for maximum coupling to the cavity. In all CBO-HF calculations performed in this work, we fulfilled the zero transverse electric field condition by minimizing the CBO-HF energy with respect to the photon displacement coordinates.^14,18,50^ We use an artificially increased coupling strength *λ*_0_ in the range of 0.001 to 0.05 au, which corresponds to effective mode volumes, see eq 3, in the single-molecule case as large as 125.27 nm^3^ (for *λ*_0_ = 0.001 au) or as small as 1.00 nm^3^ (for *λ*_0_ = 0.05 au). For all spectra shown, the underlying signals are broadened by a Lorentzian function with a width of 10 cm^–1^. All calculations were performed in a reproducible environment using the Nix package manager together with NixOS-QChem^60^ (commit f5dad404) and Nixpkgs (nixpkgs, 22.11, commit 594ef126).

## 4 Dipole Moment and Polarizability under Vibrational Strong Coupling

First, we investigate how molecular dipole moments and polarizabilities change under VSC. We limit our discussion in this paper to the results for a single CO molecule and small ensembles of CO molecules. However, to complete the picture, additional results for LiH, CO_2_, and H_2_O can be found in Section S1 of the Supporting Information. Since in the case of CO, the sign of the molecular dipole moment is wrong at the Hartree–Fock level of theory beyond a minimal basis set^51^ we only use the magnitude of the dipole moment vector and not its direction. To quantify the effect on the static polarizability, we discuss the mean polarizability *α̅* determined using the eigenvalues of the polarizability tensor ⟨ ***α*** ⟩_CBO_.

Let us first discuss how the size and structure of the basis set affect the dipole moments and polarizabilities under VSC. For this purpose, a single CO molecule and the correlation-consistent basis sets, developed by Dunning and co-workers,^58,61^ are used in the standard and augmented versions up to quadruple-zeta size. The magnitude of the dipole moment |*μ*| and the mean polarizability *α̅* (determined with ⟨ ***α*** ⟩_CBO_^num^) as well as their difference Δ|*μ*| and Δ *α̅* with respect to the field-free case are shown in Figure 1 for different basis sets as a function of *λ*_*c*_. The values ⟨ ***α*** ⟩_CBO_^num^ are determined using a field strength of 0.00001 au (0.00514 V nm^–1^).

**Figure 1:**
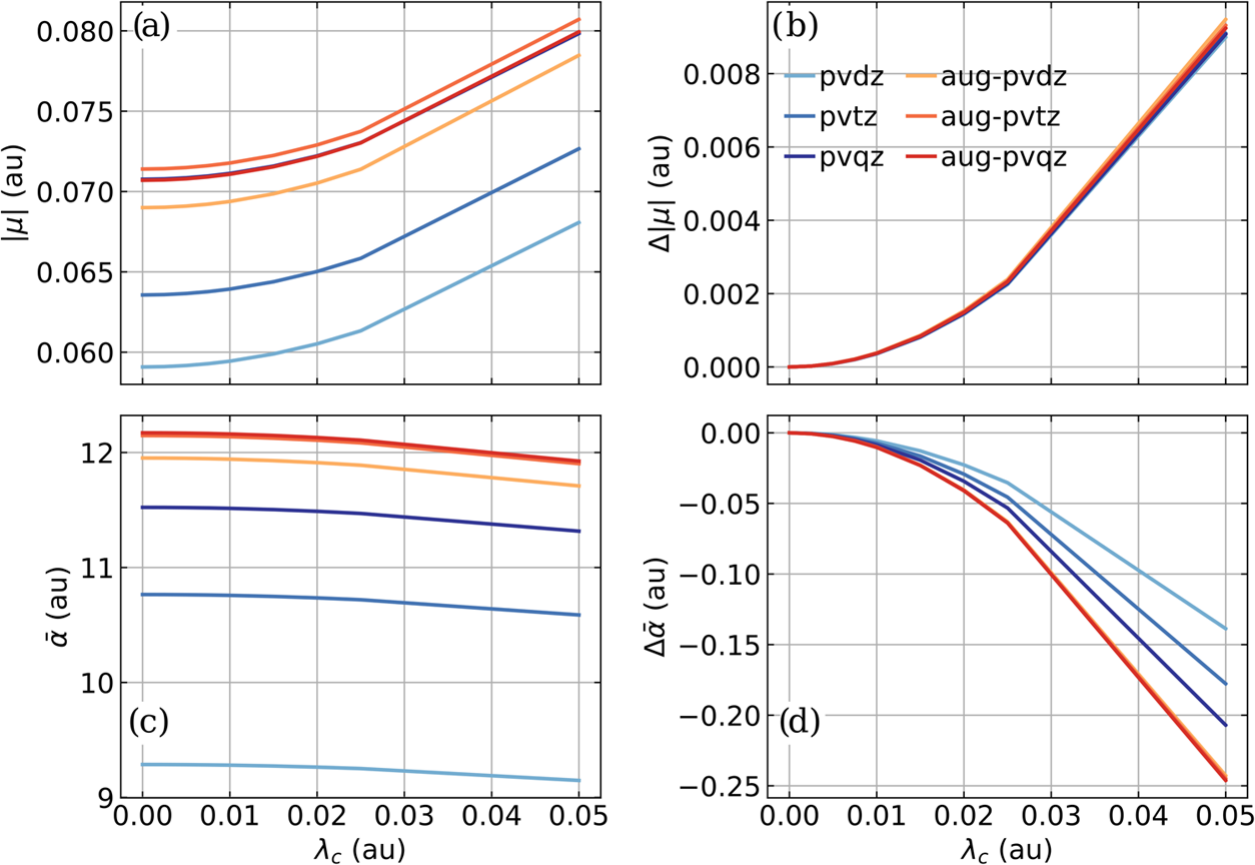
(a) Magnitude of the permanent dipole moment |*μ*| and (b) change in magnitude Δ|*μ*| for a single CO molecule as a function of cavity coupling strength *λ*_*c*_ for different basis sets (color coded). (c) Average polarizability *α̅* and (d) its change Δ *α̅* for a single CO molecule as a function of *λ*_*c*_ for different basis sets. The frequency ω_*c*_ of the single cavity mode is resonant with the fundamental transition of the CO stretching mode, and the maximum coupling strength *λ*_*c*_ corresponds to an electric vacuum field strength of 1.9 V nm^–1^ in a Fabry–Pérot-type cavity.

Qualitatively, the same trends of |*μ*| (Figure 1a) and *α̅* (Figure 1c) are observed for all basis sets with increasing *λ*_*c*_. The dipole moment increases slightly and the polarizability decreases in agreement with our previous work.^38^ The same general trends are observed for both molecular properties in the case of LiH, CO_2_, and H_2_O, which are shown in Figures S1–S3 in the Supporting Information. The absolute values of |*μ*| and *α̅* converge with an increasing basis set size. In general, the values for the augmented basis sets are more similar and converge significantly faster for *α̅*. Consequently, it seems reasonable that at least aug-cc-pVDZ should be used to determine both |*μ*| and *α̅*, since it partially outperforms the standard correlation-consistent basis sets. The effect of VSC on the dipole moment is visualized as Δ|*μ*| in Figure 1b and shows very little dependence on the chosen basis set. Only for a coupling strength *λ*_*c*_ greater than 0.03 au, some differences between the basis sets can be noticed. In the case of Δ *α̅*, the dependence on the base set is more pronounced, and the main difference is observed between the standard and the augmented versions. The augmented basis sets describe the effect of VSC on the polarizability almost identically, while the non-augmented basis set versions underestimate the effect but tend to converge to the same result with increasing size. Note that for LiH and H_2_O the dependence of |*μ*| on the basis set is more pronounced since the corresponding dipole moments are per se larger than in the case of CO.

To validate the CPHF implementation of the polarizability ⟨ ***α*** ⟩_CBO_^cphf^, we compare the two non-degenerate eigenvalues of the polarizability tensor with and without the DSE two-electron integrals (eqs 16 and 18) with respect to the formally exact ⟨ ***α*** ⟩_CBO_^num^ values. This comparison is shown in Figure 2 for the case of a single CO molecule resonantly coupled to a single cavity mode. The values ⟨ ***α*** ⟩_CBO_^num^ are determined using a field strength of 0.00001 au (0.00514 V nm^–1^).

**Figure 2:**
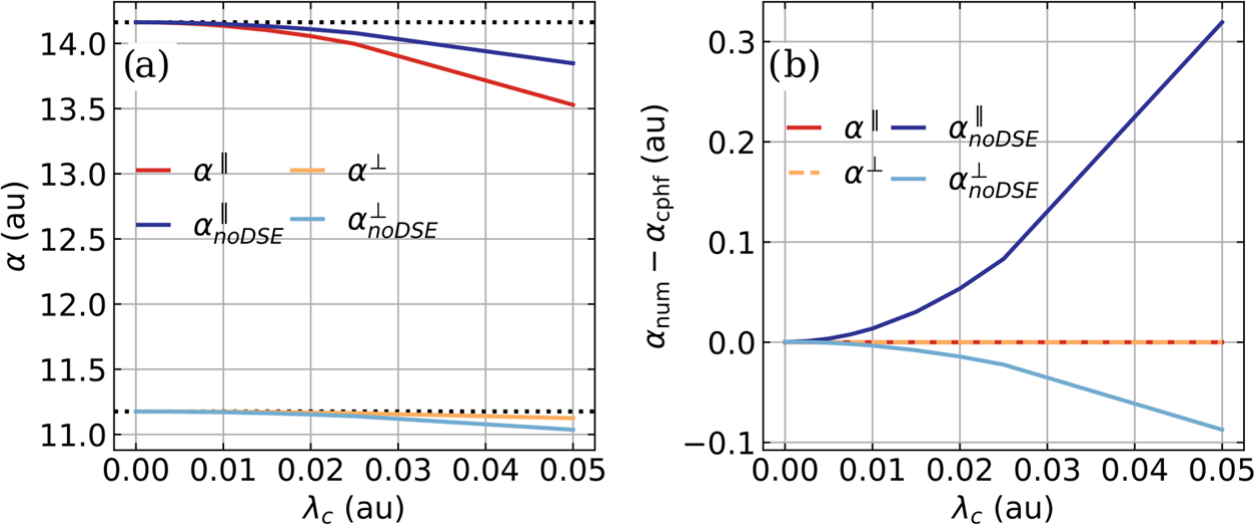
(a) Two non-degenerate eigenvalues of the polarizability tensor ⟨ ***α*** ⟩_CBO_^cphf^ of CO as a function of the cavity coupling strength *λ*_*c*_, once calculated including the DSE two-electron contributions (red and yellow) and once without it (dark and light blue). The black-dashed lines represent the corresponding field-free eigenvalues. (b) Difference between the CPHF polarizability eigenvalues (with and without DSE two-electron contributions) and the ⟨ ***α*** ⟩_CBO_^num^ eigenvalues. The frequency ω_*c*_ of the single cavity mode is resonant with the fundamental transition of the CO stretching mode, and the maximum coupling strength *λ*_*c*_ corresponds to an electric vacuum field strength of 1.9 V nm^–1^ in a Fabry–Pérot-type cavity. All values were calculated on the CBO-HF/aug-cc-pVQZ level of theory.

Regardless of whether the DSE two-electron contributions are included or not, the same trends are observed for the larger eigenvalue *α* ^∥^ and the smaller degenerate eigenvalues *α* ^⊥^ of the polarizability tensor with increasing coupling strength, see Figure 2a. Both eigenvalues of ⟨ ***α*** ⟩_CBO_ decrease with increasing *λ*_*c*_. In our simulations, the principal axis of *α* ^∥^ is aligned with the molecular and cavity polarization axis. Consequently, *α* ^∥^ is more affected by the coupling to the cavity mode than by the two degenerate eigenvalues *α* ^⊥^. Interestingly, the effect of the DSE contribution is different for *α* ^∥^ and *α* ^⊥^. For *α* ^∥^, the decrease is stronger with DSE contributions included, while for *α* ^⊥^, the opposite effect is observed. We can report similar effects for LiH, but not for CO_2_, as shown in Figures S4 and S5 in the Supporting Information. The comparison of the two types of ⟨ ***α*** ⟩_CBO_^cphf^ eigenvalues with the corresponding numerically determined eigenvalues is shown in Figure 2b. The ⟨ ***α*** ⟩_CBO_^cphf^ values, including the DSE contributions (red and yellow lines), are identical to the ⟨ ***α*** ⟩_CBO_^num^ values. Neglecting the DSE contributions (blue lines) leads to a deviation from the numerical results with an increasing coupling strength.

We now turn to possible collective effects on these properties within small molecular ensembles. In a recent publication by Horak et al.,^39^ a local change in molecular polarizability induced by collective strong coupling was observed in a simple harmonic model. In order to determine such a local effect on both the dipole moment and the polarizability, we calculate the changes per molecule as a function of the number of molecules and the coupling strength *λ*_0_

24 $$\Delta \left|\mu \left(N_{\mathrm{mol}},\lambda_{0}\right)\right|=\frac{\left|\mu_{N_{\mathrm{mol}}}\left(\lambda_{c}\right)\right|}{N_{\mathrm{mol}}}-\left|\mu_{1}\left(\lambda_{0}\right)\right|$$

25 $$\Delta \mathrm{\alpha ̅}\left(N_{\mathrm{mol}},\lambda_{0}\right)=\frac{{\mathrm{\alpha ̅}}_{N_{\mathrm{mol}}}\left(\lambda_{c}\right)}{N_{\mathrm{mol}}}-{\mathrm{\alpha ̅}}_{1}\left(\lambda_{0}\right)$$

Note that the collective coupling strength *λ*_*c*_ for the molecular ensembles is kept constant by the rescaling of *λ*_0_ (see eq 3), and the local coupling per molecule decreases as the number of molecules increases. In a situation without cavity-induced collective effects, the dipole moment and the mean polarizability per molecule would be the same as in the single molecule case, |*μ*_1_ (*λ*_0_)| and *α̅*_1_ (*λ*_0_), due to the rescaling of the collective coupling strength. Consequently, Δ|*μ*(*N*_*mol*_, *λ*_0_)| and Δ *α̅* (*N*_*mol*_, *λ*_0_) would be exactly zero. Figure 3 shows the change in the dipole moment per molecule Δ|*μ*(*N*_*mol*_, *λ*_0_)| and the change in the mean polarizability per molecule Δ*α̅* (*N*_*mol*_, *λ*_0_) for different values of *λ*_0_ as a function of the number of CO molecules. Additional results for LiH, CO_2_, and H_2_O are shown in the Supporting information Figures S6–S8.

**Figure 3:**
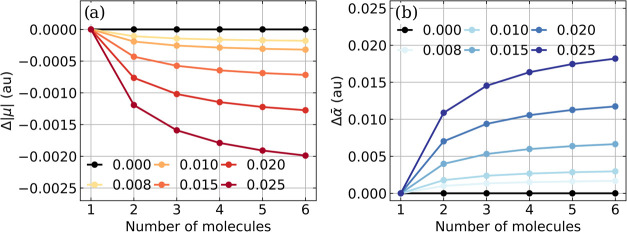
(a) Change in the dipole moment per molecule Δ|*μ*(*N*_*mol*_, *λ*_0_)| and (b) change in the mean polarizability per molecule Δ *α̅* (*N*_*mol*_, *λ*_0_) as a function of the number of CO molecules for different values of *λ*_0_ (color coded). The frequency ω_*c*_ of the single cavity mode is resonant with the fundamental transition of the CO stretching mode, and the maximum coupling strength *λ*_*c*_ corresponds to an electric vacuum field strength of 0.95 V nm^–1^ in a Fabry–Pérot-type cavity. All values were calculated on the CBO-HF/aug-cc-pVDZ level of theory.

As shown in Figure 3, both Δ|*μ* (*N*_*mol*_, *λ*_0_)| and Δ *α̅* (*N*_*mol*_, *λ*_0_) are nonzero in our simulations, regardless of the chosen coupling strength *λ*_0_. The change in the dipole moment per molecule Δ|*μ*| (Figure 3a) decreases with increasing number of molecules in the ensemble and approaches a constant nonzero value that decreases with increasing *λ*_0_. The collective interaction via the cavity mode reduces the change in dipole moment expected from the single-molecule case. Interestingly, the exact same behavior can be found for all molecules studied in this work, see Figures S6–S8 in the Supporting Information, indicating that this collective effect is rather general. Note that the only exception is CO_2_, which has no permanent dipole moment, and the coupling strength used is not large enough to induce a relevant dipole moment in the system. The change in the mean polarizability per molecule Δ *α̅*, shown in Figure 3b, increases with the number of molecules in the ensemble and approaches a constant nonzero value that increases with increasing *λ*_0_. This result indicates that cavity-mediated collective interactions increase the polarizability per molecule in comparison with the single-molecule case. Again, this trend seems quite general, as it is also observed for LiH, CO_2_, and H_2_O, as shown in Supporting Information Figures S6–S8. For all molecules studied, the change per molecule for both properties with respect to *N*_*mol*_ is proportional to 1 – 1/*N*_*mol*_ and approaches a constant, nonzero value that depends on the coupling strength. We had already observed the same scaling behavior for the DSE-induced intermolecular dipole–dipole energy contribution for small ensembles of hydrogen fluoride molecules.^18^ We consider the fact that we observe a collective effect on both dipole moment and polarizability under VSC to be one of the central findings of this work, in line with the results obtained previously for an analytic harmonic model.^39^ These collective effects clearly indicate that a single molecule in the cavity-coupled ensemble is not independent of the rest, even if the corresponding modifications are small.

## 5 Vibro-Polaritonic Spectra for Formaldehyde

Apart from being an important molecular property for understanding molecular interactions, such as the London dispersion forces,^35,36^ the polarizability also determines how molecules interact with light. In this work, we combine the static polarizability with our work on vibro-polaritonic spectra in the harmonic approximation^28^ to calculate Raman activity and scattering factors under VSC. As a first demonstration and also to investigate the different effects of VSC on IR and Raman spectra, we simulate both vibro-polaritonic spectra of a single formaldehyde molecule coupled to two cavity modes of orthogonal polarization with the same frequency, effectively modeling a simplified Fabry–Pérot-like setup. The single formaldehyde molecule is oriented with respect to the center of the nuclear charges, the molecular plane is in the *x*–*y* plane, and the carbonyl group is aligned with the *y-*axis of the laboratory frame, as shown in the inset of Figure 4. The polarization axes of the two cavity modes are aligned with the *x-axis* and the *y-axis*, respectively. Formaldehyde has six vibrational degrees of freedom: three bending modes, one out-of-plane and two in-plane, the CO stretching mode, and one symmetric and one asymmetric CH_2_ stretching mode. The symmetric and asymmetric CH_2_ stretching modes are both IR and Raman active and are reasonably close in energy with Δ*ν* ≈ 80 cm^–1^. The transition dipole moment for the symmetric mode points along the *y-*axis and that for the asymmetric mode along the *x-axis*. Therefore, we choose the cavity frequencies ω_*c*_ to be larger than 3000 cm^–1^ to focus the discussion on the spectral range of these two stretching modes. The bare molecular vibronic IR and Ramana spectra and the vibro-polaritonic IR and Ramana spectra of a single formaldehyde molecule coupled to two cavity modes are shown in Figure 4 for two different cavity frequencies.

**Figure 4:**
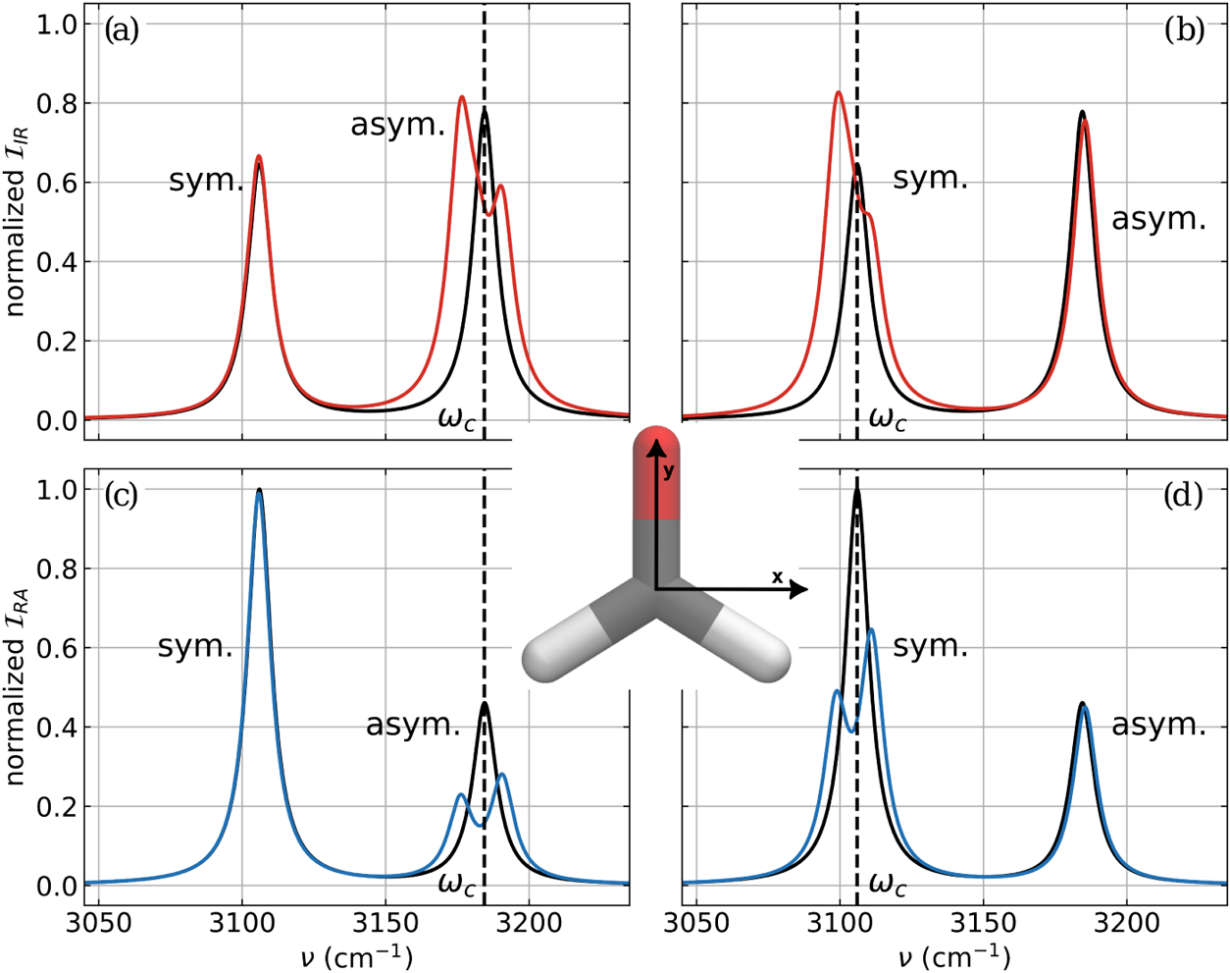
(a, b) Vibro-polaritonic IR spectra shown in red and (c, d) vibro-polaritonic Raman spectra shown in blue of a single formaldehyde molecule coupled to two cavity modes. Only the frequency range above 3000 cm^–1^ for the symmetric and asymmetric CH_2_ stretching modes is shown. For (a) and (c), the cavity modes are resonant with the asymmetric stretching and for (b) and (d) with the symmetric stretching; the corresponding frequencies ω_*c*_ are marked with black-dashed lines. The spectra are normalized with respect to the maximum intensity of the cavity-free spectrum, shown in black. The molecular orientation with respect to the two cavity polarization axes *x* and *y* is shown in the middle inset. All spectra are calculated at the CBO-HF/aug-cc-pVDZ level of theory using a coupling strength *λ*_*c*_ of 0.010 au, which corresponds to an electric vacuum field strength of 0.44 V nm^–1^ if the cavity is resonant to the asymmetric stretching mode and 0.43 V nm^–1^ if the cavity is resonant to the symmetric stretching mode in a Fabry–Pérot-type cavity.

Let us start by analyzing the vibro-polaritonic IR spectra shown in Figure 4a,b. The asymmetric stretching mode (*ν* = 3184 cm^–1^ HF/aug-cc-pVDZ level of theory) is more intense than the symmetric stretching mode (*ν* = 3106 cm^–1^ HF/aug-cc-pVDZ level of theory) for the spectrum of the uncoupled formaldehyde molecule. As expected, the splitting into an LP and a UP transition is observed for the symmetric/asymmetric stretching mode when coupled resonantly to the cavity. In both cases, the LP transition is broadened by the presence of the second cavity mode; the reason for this will be discussed later. In the case of resonant coupling to the symmetric stretching mode (Figure 4b), this leads to the situation that the UP transition is only visible as a shoulder for the chosen coupling strength and broadening of the spectrum. The combined intensity of the LP and UP transition is larger than the intensity of the uncoupled molecular transition, which is reasonable given our observation that the dipole moment itself increases under VSC. For the chosen coupling strength of 0.010 au, the non-resonant transition is not affected in terms of both intensity and frequency. In agreement with the literature^20,28,46,62,63^ the formed pair of vibro-polaritonic transition is asymmetric in two ways. First, the Rabi splitting *Ω*_*R*_ between LP and UP is asymmetric, where LP is more red-shifted than UP is blue-shifted with respect to ω_*c*_. This asymmetry can be understood as a detuning and a change in optical length of the cavity when interacting with the molecule.^20,28,63^ Second, the signal intensity is asymmetric, with the LP transition being more intense than the UP transition, which has also been observed in the literature.^46,62^ Recently, Huang and Liang^46^ explained this asymmetry based on a simplified Hessian model. They argue that the transition dipole moment of the LP state is formed by the positive linear combination of the dipole outside the cavity and a cavity-induced dipole moment, while the corresponding UP dipole moment is formed by the negative linear combination, leading to a higher intensity for the LP transition and a lower intensity for the UP transition.

Since both vibro-polaritonic IR spectra and Raman spectra are based on the same Hessian matrix, we only discuss the Raman intensities of the vibro-polaritonic transitions shown in Figure 4c,d. In the case of uncoupled formaldehyde, the symmetric stretching mode (*ν* = 3106 cm^–1^) is more intense than the asymmetric stretching mode (*ν* = 3184 cm^–1^). When resonantly coupled to the cavity modes, we can also observe the splitting into an LP and a UP transition in the vibro-polaritonic Raman spectra. Note that this is only possible because both CH_2_ stretching modes are IR and Raman active; otherwise, there would be no formation of polaritonic states, i.e., the transition is not IR active, or they would not be observable in the Raman spectrum. In contrast to the corresponding IR spectra, the Raman intensities of the LP and UP transitions are significantly lower than those for the bare molecular transition. This can be related to our observation that the polarizability itself decreases under VSC. In agreement with Huang and Liang^46^ we observe an asymmetry in the intensities of the LP and UP transitions. In contrast to the vibro-polaritonic IR spectra, the UP transition is more intense compared to the LP transition in the Raman spectra.

In Figure 5, the dispersion with respect to cavity frequency ω_*c*_ for both the vibro-polaritonic IR spectrum and the vibro-polaritonic Raman spectrum is shown using the same parameters as previously used in Figure 4. The dispersion curves are obtained by scanning ω_*c*_ for both cavity modes in the range from 3030 cm^–1^ to 3260 cm^–1^ with a step size of 0.5 cm^–1^.

**Figure 5:**
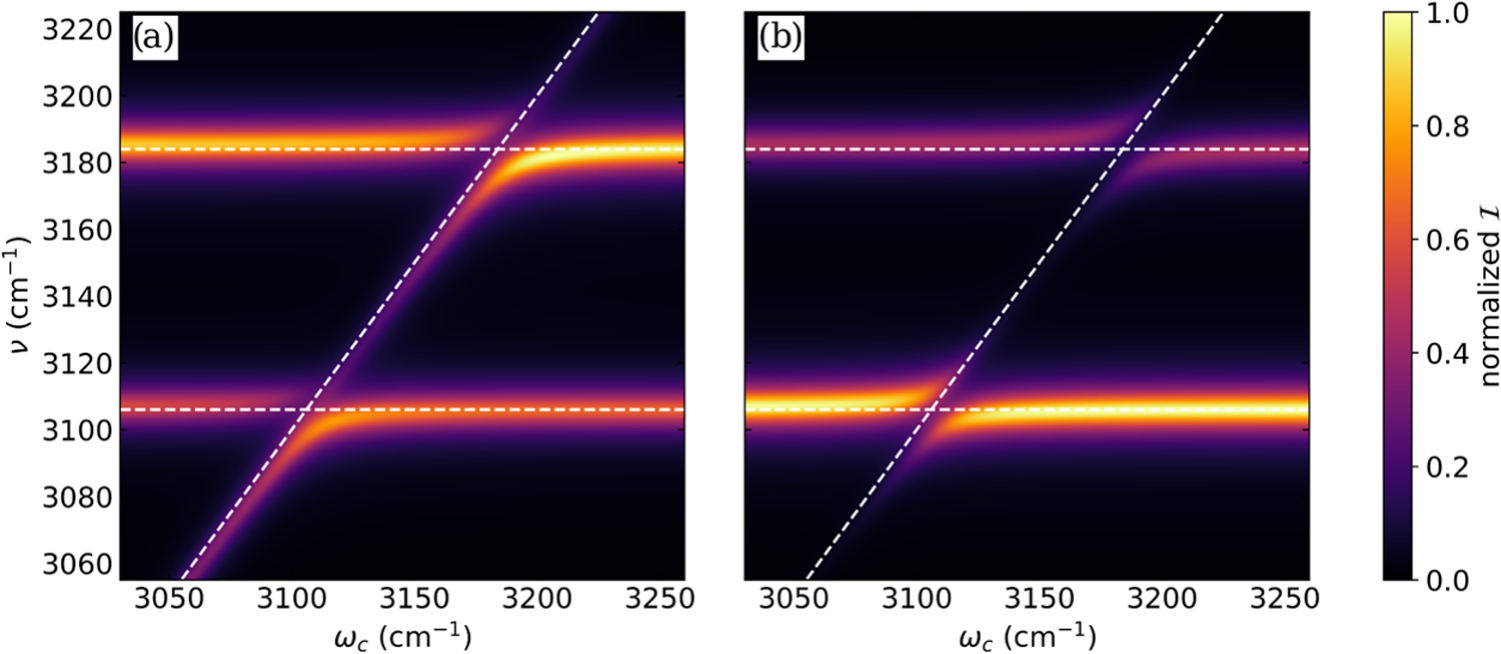
Normalized dispersion with respect to cavity frequency ω_*c*_ of (a) the vibro-polaritonic IR spectrum and (b) the vibro-polaritonic Raman spectrum of a single formaldehyde molecule for different cavity frequencies. The horizontal white dashed lines indicate the bare molecule transitions, and the diagonal white dashed line indicates the bare cavity frequency. All underlying spectra are calculated at the CBO-HF/aug-cc-pVDZ level of theory using a coupling strength *λ*_*c*_ of 0.010 au, which corresponds to values of the electric vacuum field strength in the range of range 0.43 to 0.44 V nm^–1^ in a Fabry–Pérot-type cavity.

In the vicinity of the two resonances (around 3106 and 3184 cm^–1^, i.e., close to zero detuning of the cavity), the formation of the LP and the UP leads to the appearance of the well-known avoided crossing pattern in the dispersion curves of both spectra, shown in Figure 4a,b. As detuning increases to smaller or larger values of ω_*c*_, the observed vibro-polaritonic transitions become more molecular or photonic. The more photonic transition loses intensity, while the more molecular transitions remain visible and constant in frequency. When the dispersion curves of the IR and Raman spectra are compared, the more photonic transition loses its Raman intensity much faster than its IR intensity. Another difference between the IR and Raman signals is observed at larger detunings. A direct comparison of the IR intensities at ω_*c*_ = 3030 cm^–1^ (far left) and ω_*c*_ = 3260 cm^–1^ (far right) for the signal corresponding to the symmetric stretching mode (*ν* = 3180 cm^–1^) clearly shows that the intensity increases for larger ω_*c*_, even though the transition is no longer resonantly coupled. In contrast, the Raman intensities of the out-of-resonance molecular transitions remain approximately the same before and after hybridization. In addition, the discussed decoupling of molecular and photonic transitions is also clearly visible in the |*a*_*c*_|^2^ values shown in Figure S9 of the Supporting Information. In the CBOA, the normal mode vectors have terms *a*_*c*_ describing the change in the classical photon displacement field coordinates *q*_*c*_^(*x*)^ and *q*_*c*_^(*y*)^. The value of |*a*_*c*_|^2^ for a given normal mode is a measure of how strongly the corresponding vibrational transition interacts with the photon field and allows us to estimate the photonic character of the corresponding transitions.^28^

The last aspect we want to discuss is how both vibro-polaritonic IR spectra and Raman spectra change with increasing coupling strength *λ*_*c*_ for the case of a single formaldehyde molecule coupled to two cavity modes resonant with the asymmetric CH_2_ stretching mode. The spectra for different values of *λ*_*c*_ are shown in Figure 6, and the |*a*_*c*_|^2^ values for all four relevant transitions (sorted by ascending frequency and labeled with Latin numbers) as a function of *λ*_*c*_ are shown in Figure 7. For *λ*_*c*_ = 0.005 au, the light-matter coupling is strong enough to observe the formation of the LP transition and the UP transition, and two strongly overlapping signals are visible. As discussed above, the LP transition is more intense in the IR spectrum, and the UP transition is more intense in the Raman spectrum. The pair LP and UP is formed as expected by the asymmetric stretching mode and the cavity mode with the *x* polarization axis, see Figure 7a, where both transitions are labeled II and IV. The non-resonant symmetric stretching mode is not altered by the cavity interaction. As the coupling strength increases, the Rabi splitting Ω_*R*_ between the II and IV transitions increases. The UP transition (IV, highest frequency signal) is blue-shifted and loses IR intensity. In contrast, its Raman intensity remains constant with increasing *λ*_*c*_. The corresponding LP transition (II) is more red-shifted and gains IR intensity while losing Raman intensity. At the same time, an additional transition becomes more pronounced in the IR spectrum between the LP and UP transitions, starting as a shoulder of the LP signal for *λ*_*c*_ = 0.010 au. In the Raman spectra, this transition is only observable as a very weak signal around 3160 cm^–1^ for the highest coupling strength studied, see Figure 6b. Analyzing the corresponding |*a*_*c*_|^2^ values shown in Figure 7b, it becomes clear that this transition originates from the cavity photon mode with the *y* polarization axis, labeled III. This transition is mostly photonic for all coupling strengths. However, with increasing coupling strength, this cavity photon mode can weakly hybridize with the off-resonant symmetric stretching mode, labeled I in Figure 7, leading to a strong red shift of the cavity mode and an increase in intensities. This mixing also affects the symmetric stretching mode for coupling strengths larger than 0.020 au. The mostly molecular mode is also slightly red-shifted, while gaining IR intensity and losing Raman intensity. This off-resonant hybridization leads to the observed broadening of the LP signal at lower coupling strengths, regardless of whether the cavity is resonant with the symmetric or asymmetric stretching mode, as already mentioned in the discussion of Figure 4. In summary, for the higher coupling strengths, we observe some kind of nested polaritons that can be understood as two pairs of LP and UP transitions. These nested polariton features, due to interactions with off-resonant cavity modes, are also observed in VSC experiments^64,65^ in the strong and ultra-strong coupling regime. However, it should be noted that both experiments are not fully comparable since they are based on collective strong coupling in the presence of multiple (more than two) cavity modes.

**Figure 6:**
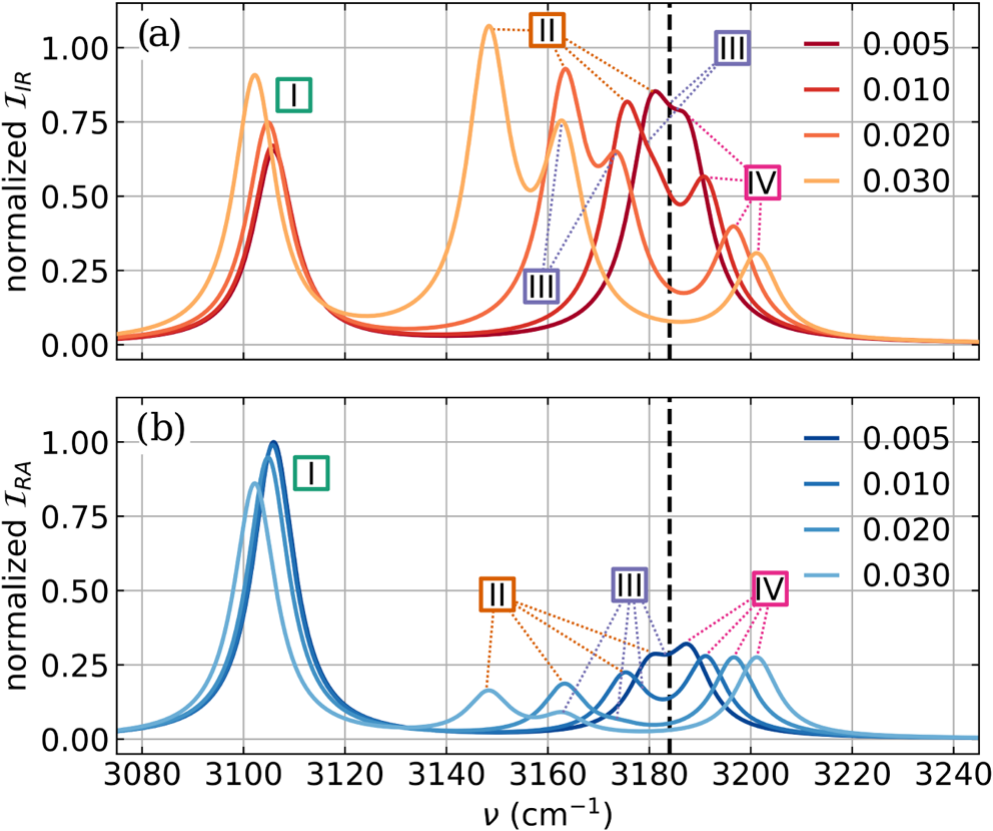
(a) Vibro-polaritonic IR spectra and (b) vibro-polaritonic Raman spectra of a single formaldehyde molecule for increasing coupling strength *λ*_*c*_. The cavity frequency is chosen to be resonant with the asymmetric CH_2_ stretching mode of 3184 cm^–1^ and indicated as a black-dashed line. The maximum coupling strength *λ*_*c*_ corresponds to an electric vacuum field strength of 1.3 V nm^–1^ in a Fabry–Pérot-type cavity.

**Figure 7:**
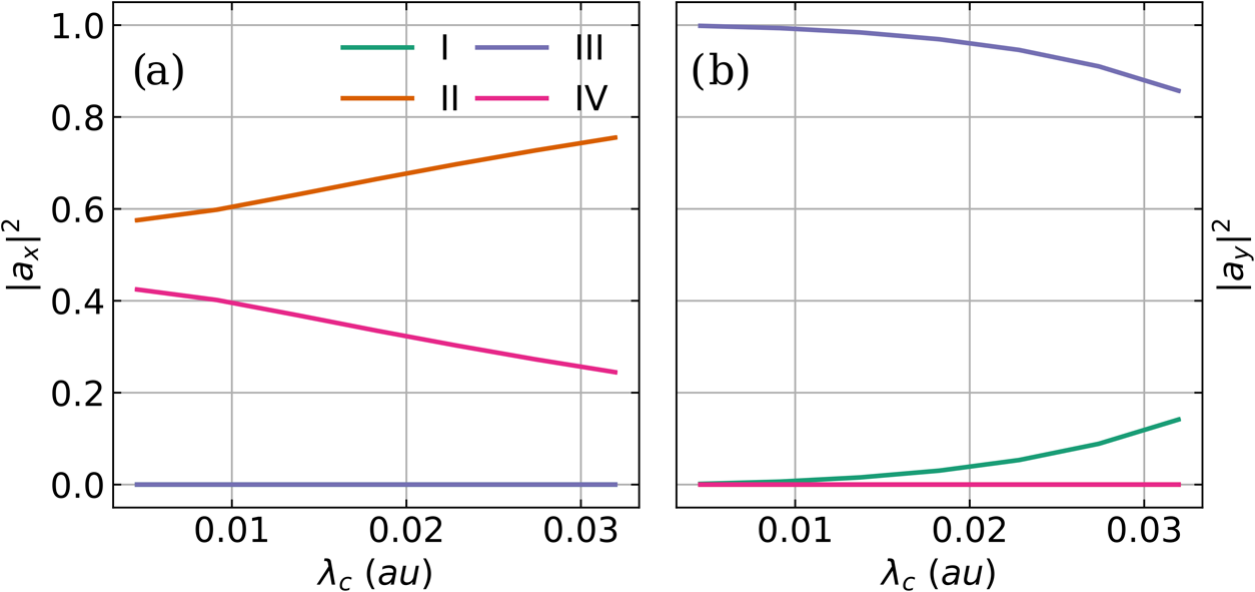
|*a*_*c*_|^2^ values of the four relevant vibro-polaritonic transitions (sorted by ascending frequency and labeled with Latin numbers) corresponding to (a) the cavity mode with *x* polarization and (b) the cavity mode with *y* polarization as a function of *λ*_*c*_. The underlying normal modes are calculated at the CBO-HF/aug-cc-pVDZ level of theory, and the coupling strength *λ*_*c*_ increases from 0.005 to 0.033 au. The cavity frequency ω_*c*_ of 3184 cm^–1^ resonates with the asymmetric stretching mode. The maximum coupling strength *λ*_*c*_ corresponds to an electric vacuum field strength of 1.3 V nm^–1^ in a Fabry–Pérot-type cavity.

## 6 Summary and Conclusions

We have derived an analytic formulation of the static polarizability for the cavity Born–Oppenheimer Hartree–Fock ansatz^18^ using linear-response theory. The obtained polarizabilities have been used to calculate the vibro-polaritonic Raman spectra under VSC in the harmonic approximation using a wave-function-based methodology. By studying the effect of VSC for different molecules (CO, LiH, CO_2_ and H_2_O) in the single molecule case, we were able to generalize our previous observations^38^ that the permanent dipole moment increases while the static polarizability decreases with increasing coupling strength *λ*_*c*_. These two trends were observed for all four molecules studied, but for the case of CO_2_ without a permanent dipole moment, the VSC does not induce a significant dipole moment for the coupling strength used in this work. One of our main objectives in this work was to investigate whether a cavity-mediated local modification of ***α*** under VSC, as demonstrated for a full-harmonic model,^39^ exists when describing real molecular ensembles. By calculating the mean polarizability per molecule for ensembles of CO, LiH, CO_2_, and H_2_O molecules under a VSC, we observed a cavity-mediated local modification. The change per molecule is proportional to 1 – 1/*N*_*mol*_ and approaches a constant, nonzero value that depends on the coupling strength. We had already observed the same scaling behavior for the DSE-induced intermolecular dipole–dipole energy contribution.^18^ A similar trend is observed for the change in the magnitude of the permanent dipole moment per molecule in such ensembles. These per-molecule changes in both molecular properties can be interpreted as a situation in which the single molecule in the cavity-coupled ensemble is no longer independent of the rest.

When simulating Raman spectra using Raman activities based on the harmonic approximation for a single formaldehyde molecule coupled to two cavity modes, we observed similar effects of VSC as Huang and Liang.^45,46^ The Raman intensities for LP and UP are asymmetric but otherwise behave differently compared to the corresponding IR intensities. The Raman signal of the UP transition is more intense than the LP signal, and overall, the signals weaken with increasing coupling strength. Although these trends are present in our theoretical description, we are unable to provide a comprehensive explanation, and a more in-depth study of vibro-polaritonic Raman spectra is needed. However, we would like to emphasize that due to the Fabry–Pérot-like setup and the chosen molecular system, we are able to observe nested polariton features in the IR and Raman spectra due to interactions with off-resonant cavity modes, which are also observed in VSC experiments.^64,65^

## Supplementary Material



## Notes

See the supplementary material for additional figures showing the dipole moments and polarizabilities of LiH, CO_2_, and H_2_O under vibrational strong coupling and the vibro-polaritonic normal mode analysis for formaldehyde. All data underlying this study are available from the corresponding author upon reasonable request (PDF)
