# Supplementary material for: Molecular Polarizability under Vibrational Strong Coupling
Source: J Chem Theory Comput. 2025 May 14;21(10):5171–81. doi: 10.1021/acs.jctc.5c00461 (PMC12120982; doi:10.1021/acs.jctc.5c00461)
Supplement: Supplementary file 1 [file ct5c00461_si_001.pdf]

Supporting Information: Molecular Polarizability under Vibrational Strong Coupling

Thomas Schnappinger* and Markus Kowalewski*

Department of Physics, Stockholm University, AlbaNova University Center, SE-106 91 Stockholm, Sweden

E-mail: thomas.schnappinger@fysik.su.se; markus.kowalewski@fysik.su.se

Contents

S1 Additional Results: Dipole Moment and Polarizability under Vibrational Strong Coupling **S3**

S2 Additional Results: Vibro-Polaritonic Spectra for Formaldehyde **S11**

References **S13**

S1 Additional Results: Dipole Moment and Polarizability under Vibrational Strong Coupling

Since we limited the discussion in the manuscript to the results of a single CO molecule and small ensembles of CO molecules, this section includes additional results for LiH, CO_2, and H_2O. These three examples represent different cases with a stronger permanent dipole moment, without a permanent dipole moment, as well as molecules with more than two atoms and a non-linear molecule.

The magnitude of the dipole moment $|\mu|$ and the mean polarizability $\bar{\alpha}$ (determined with $\langle \boldsymbol{\alpha} \rangle_{\text{CBO}}^{\text{num}}$) as well as their difference $\Delta|\mu|$ and $\Delta\bar{\alpha}$ with respect to the cavity-free case for LiH, CO_2 and H_2O are shown in Figs. S1, S2, S3 for different basis sets as a function of λ_c. The values $\langle \boldsymbol{\alpha} \rangle_{\text{CBO}}^{\text{num}}$ are determined using a field strength of 0.000 01 au (0.005 14 V nm^{-1}).

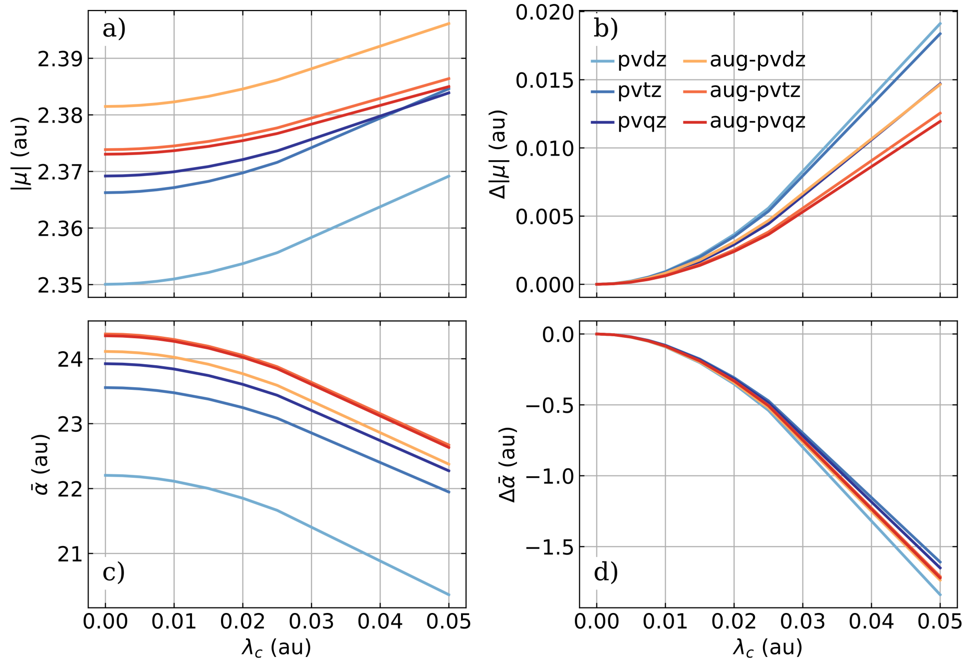

Figure S1: a) Magnitude of the permanent dipole moment $|\mu|$ and b) change in magnitude $\Delta|\mu|$ for a single LiH molecule as a function of cavity coupling strength λ_c for different basis sets (color coded). c) Average polarizability $\bar{\alpha}$ and d) its change $\Delta\bar{\alpha}$ for a single LiH molecule as a function of λ_c for different basis sets. The frequency ω_c of the single cavity mode is resonant with the fundamental transition of the LiH stretching mode and the maximum coupling strength λ_c corresponds to an electric vacuum field strength $1.465\,\mathrm{V\,nm^{-1}}$ in a Fabry-Pérot-type cavity.

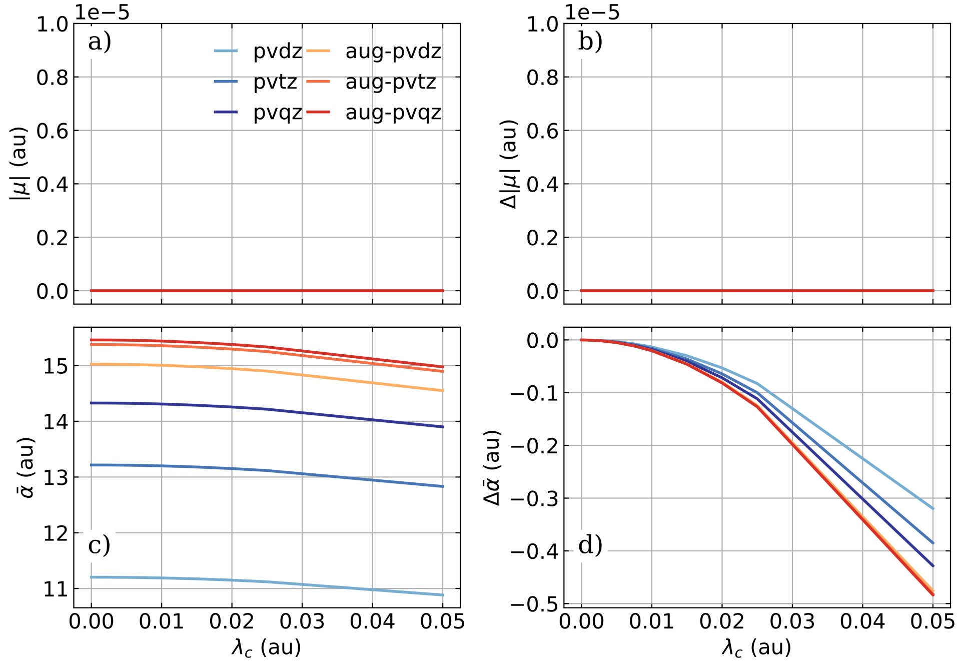

Figure S2: a) Magnitude of the permanent dipole moment $|\mu|$ and b) change in magnitude $\Delta|\mu|$ for a single CO_2 molecule as a function of cavity coupling strength λ_c for different basis sets (color coded). c) Average polarizability $\bar{\alpha}$ and d) its change $\Delta\bar{\alpha}$ for a single CO_2 molecule as a function of λ_c for different basis sets. The frequency ω_c of the single cavity mode is resonant with the fundamental transition of the CO_2 asymmetric stretching mode and the maximum coupling strength λ_c corresponds to an electric vacuum field strength $1.955\,\mathrm{V\,nm^{-1}}$ in a Fabry-Pérot-type cavity.

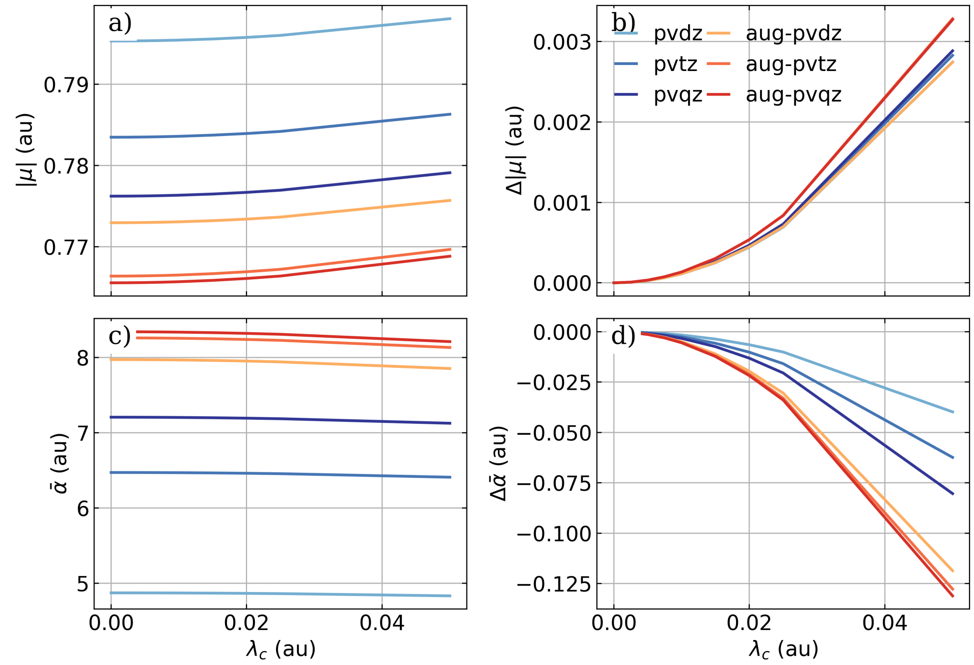

Figure S3: a) Magnitude of the permanent dipole moment $|\mu|$ and b) change in magnitude $\Delta|\mu|$ for a single H_2O molecule as a function of cavity coupling strength λ_c for different basis sets (color coded). c) Average polarizability $\bar{\alpha}$ and d) its change $\Delta\bar{\alpha}$ for a single H_2O molecule as a function of λ_c for different basis sets. The frequency ω_c of the single cavity mode is resonant with the fundamental transition of the H_2O symmetric stretching mode and the maximum coupling strength λ_c corresponds to an electric vacuum field strength of $2.525\,\mathrm{V\,nm^{-1}}$ in a Fabry-Pérot-type cavity.

Figs. S4 and S5 show the comparison between the two versions coupled-perturbed Hartree-Fock (CPHF) implementation of polarizability $\langle \boldsymbol{\alpha} \rangle_{\mathrm{CBO}}^{\mathrm{cphf}}$ and the formally exact $\langle \boldsymbol{\alpha} \rangle_{\mathrm{CBO}}^{\mathrm{num}}$ values for LiH and CO_2. The values $\langle \boldsymbol{\alpha} \rangle_{\mathrm{CBO}}^{\mathrm{num}}$ are determined using a field strength of 0.000 01 au (0.005 14 V nm^{-1}).

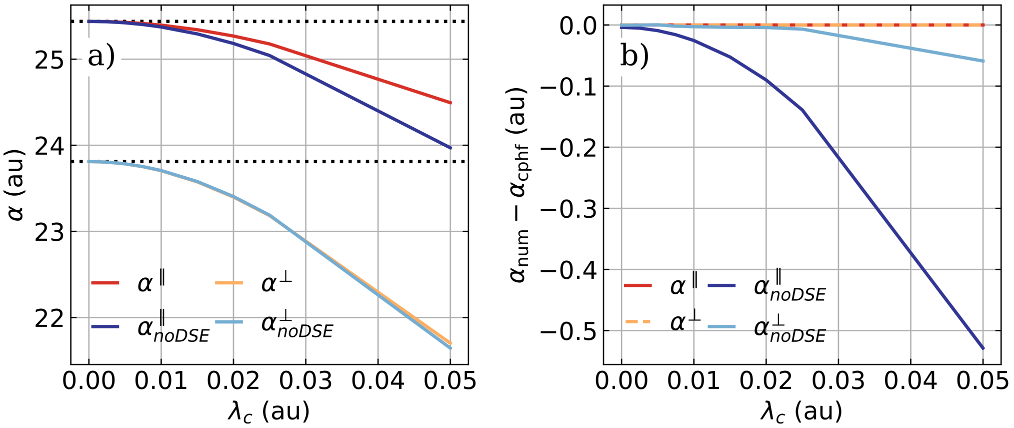

Figure S4: a) The two non-degenerate eigenvalues of the polarizability tensor $\langle \boldsymbol{\alpha} \rangle_{\mathrm{CBO}}^{\mathrm{cphf}}$ of LiH as a function of the cavity coupling strength λ_c once calculated including the dipole self-energy (DSE) two-electron contributions (red and yellow) and once without it (dark and light blue). The black dashed lines represent the corresponding field-free eigenvalues. b) The difference between the CPHF polarizability eigenvalues (with and without DSE two-electron contributions) and the $\langle \boldsymbol{\alpha} \rangle_{\mathrm{CBO}}^{\mathrm{num}}$ eigenvalues. The frequency ω_c of the single cavity mode is resonant with the fundamental transition of the LiH stretching mode and the maximum coupling strength λ_c corresponds to an electric vacuum field strength of 1.465 V nm^{-1} in a Fabry-Pérot-type cavity. All values calculated on the CBO-HF/aug-cc-pVQZ level of theory.

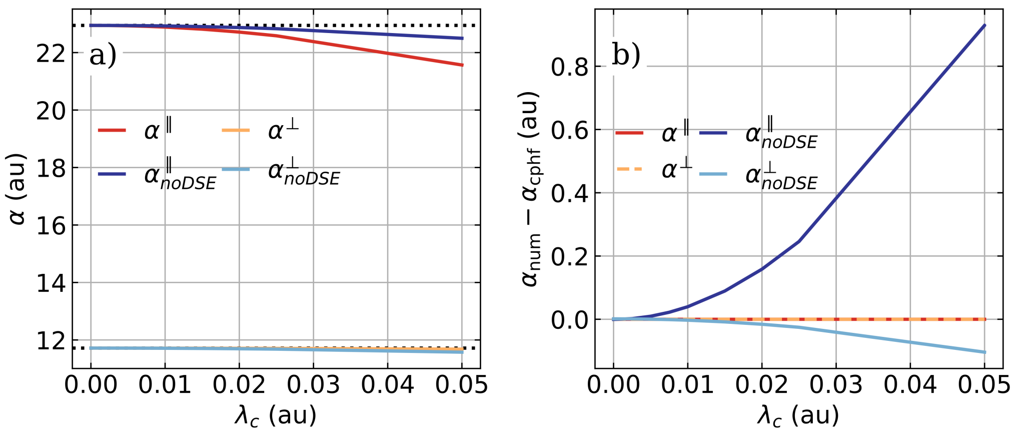

Figure S5: a) The two non-degenerate eigenvalues of the polarizability tensor $\langle \boldsymbol{\alpha} \rangle^{\mathrm{cphf}}_{\mathrm{CBO}}$ of CO_2 as a function of the cavity coupling strength λ_c once calculated including the DSE two-electron contributions (red and yellow) and once without it (dark and light blue). The black dashed lines represent the corresponding field-free eigenvalues. b) The difference between the CPHF polarizability eigenvalues (with and without DSE two-electron contributions) and the $\langle \boldsymbol{\alpha} \rangle^{\mathrm{num}}_{\mathrm{CBO}}$ eigenvalues. The frequency ω_c of the single cavity mode is resonant with the fundamental transition of the CO_2 stretching mode and the maximum coupling strength λ_c corresponds to an electric vacuum field strength of $1.955\,\mathrm{V\,nm^{-1}}$ in a Fabry-Pérot-type cavity. All values calculated on the CBO-HF/aug-cc-pVQZ level of theory.

The change in the dipole moment per molecule $\Delta\left|\mu\left(N_{mol}, \lambda_0\right)\right|$ and the change in the mean polarizability per molecule $\Delta\bar{\alpha}\left(N_{mol}, \lambda_0\right)$ for different values of λ_0 as a function of the number of molecules for LiH, CO_2 and H_2O is shown in Figs. S6, S7 and S8

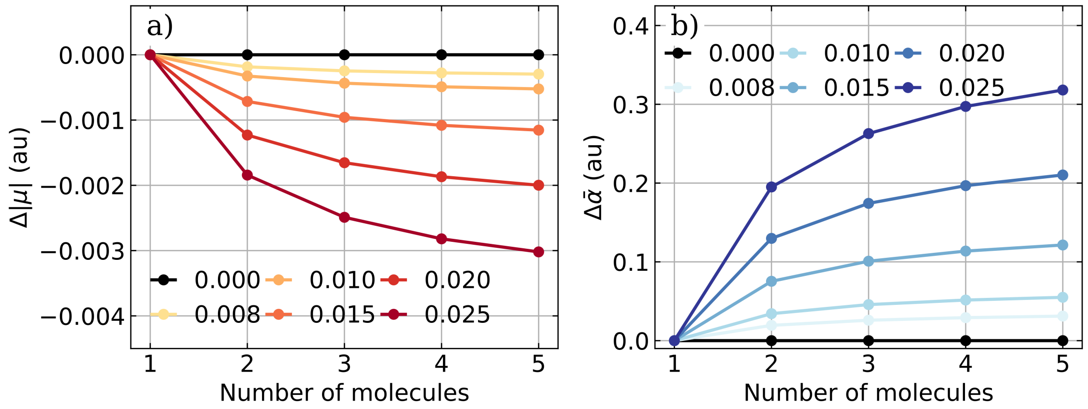

Figure S6: a) Change in the dipole moment per molecule $\Delta\left|\mu\left(N_{mol}, \lambda_0\right)\right|$ and b) change in the mean polarizability per molecule $\Delta\bar{\alpha}\left(N_{mol}, \lambda_0\right)$ as function of the number of LiH molecules for different values of λ_0 (color coded). The frequency ω_c of the single cavity mode is resonant with the fundamental transition of the LiH stretching mode and the maximum coupling strength λ_c corresponds to an electric vacuum field strength of $0.732\,\mathrm{V\,nm^{-1}}$ in a Fabry-Pérot-type cavity. All values calculated on the CBO-HF/aug-cc-pVDZ level of theory.

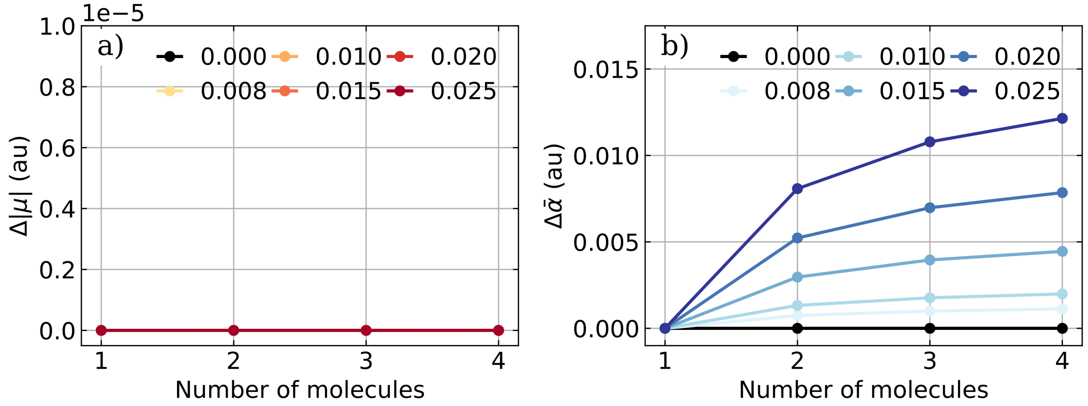

Figure S7: a) Change in the dipole moment per molecule $\Delta\left|\mu\left(N_{mol}, \lambda_0\right)\right|$ and b) change in the mean polarizability per molecule $\Delta\bar{\alpha}\left(N_{mol}, \lambda_0\right)$ as function of the number of CO_2 molecules for different values of λ_0 (color coded). The frequency ω_c of the single cavity mode is resonant with the fundamental transition of the CO_2 asymmetric stretching mode and the maximum coupling strength λ_c corresponds to an electric vacuum field strength of $0.977\,\mathrm{V\,nm^{-1}}$ in a Fabry-Pérot-type cavity. All values calculated on the CBO-HF/aug-cc-pVDZ level of theory.

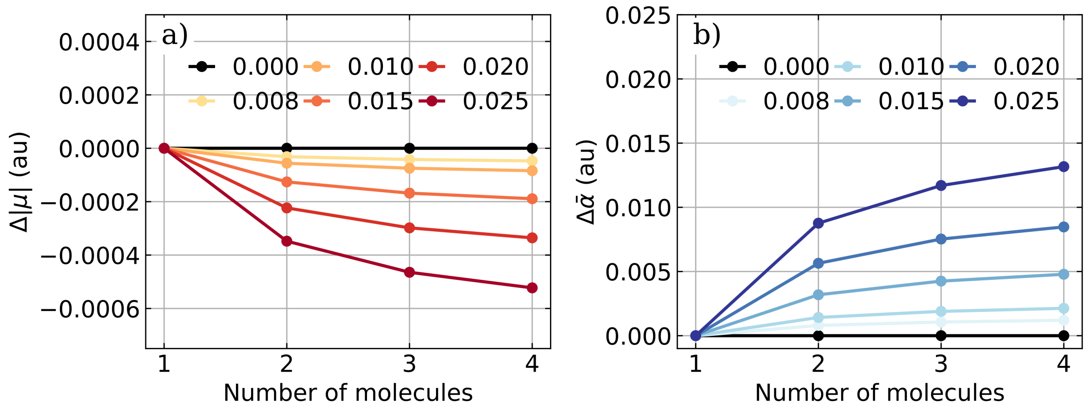

Figure S8: a) Change in the dipole moment per molecule $\Delta\left|\mu\left(N_{mol}, \lambda_0\right)\right|$ and b) change in the mean polarizability per molecule $\Delta\bar{\alpha}\left(N_{mol}, \lambda_0\right)$ as function of the number of H_2O molecules for different values of λ_0 (color coded). The frequency ω_c of the single cavity mode is resonant with the fundamental transition of the H_2O symmetric stretching mode and the maximum coupling strength λ_c corresponds to an electric vacuum field strength of $1.263\,\mathrm{V\,nm^{-1}}$ in a Fabry-Pérot-type cavity. All values calculated on the CBO-HF/aug-cc-pVDZ level of theory.

S2 Additional Results: Vibro-Polaritonic Spectra for Formaldehyde

The general concept of performing a normal mode analysis in the cavity Born-Oppenheimer approximation (CBOA) was introduced in our previous work,[1] and the reader is referred to the paper for details of the theory. For convenience, the main ideas are summarized in the following. The harmonic approximation gives access to the normal modes $\boldsymbol{Q}^k$. In the CBOA the normal mode vectors have terms a_c describing the change in the classical photon displacement field coordinates q_c. The value of $|a_c|^2$ for a given normal mode is a measure of how strongly the corresponding vibrational transition interacts with the photon field. For an uncoupled light-matter system, a pure molecular transition is characterized by a $|a_c|^2$ value of zero, whereas the bare photon mode has a value of one. Note that due to the length gauge description q_c and the corresponding value $|a_c|^2$ are no longer a pure photonic quantity if light and matter are coupled.[2–4] However, $|a_c|^2$ can still be used as a probe to identify how photonic the corresponding vibrational transition is. The information obtained is comparable to the coefficients in the Hopfield models.[5] The $|a_c|^2$ values to characterize the four relevant normal modes of a single formaldehyde molecule interacting with two orthogonal cavity modes are shown in Fig. S9 as a function of the cavity frequency in a range of $3030\,\mathrm{cm}^{-1}$ to $3260\,\mathrm{cm}^{-1}$.

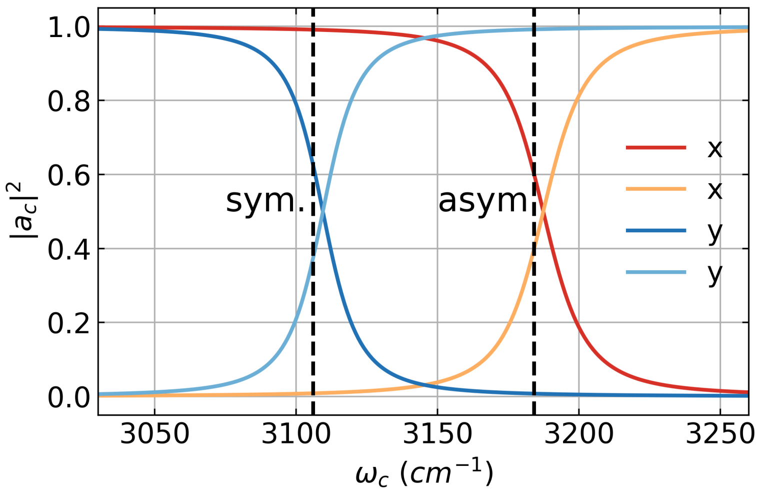

Figure S9: $|a_c|^2$ values describing the change in $q_c^{(x)}$ and $q_c^{(y)}$ for the four relevant normal modes as a function of the cavity frequency. The bluish lines correspond to the $|a_c|^2$ values for the symmetric stretching and the cavity mode with y polarization, and the reddish lines correspond to the $|a_c|^2$ values for the asymmetric stretching and the cavity mode with x polarization. The underlying normal modes are calculated at the CBO-HF/aug-cc-pVDZ level of theory using a coupling strength λ_c of $0.010\,\mathrm{au}$ which corresponds to values of the electric vacuum field strength in the range of $0.425\,\mathrm{V\,nm^{-1}}$ to $0.442\,\mathrm{V\,nm^{-1}}$ in a Fabry-Pérot-type cavity.

References

(1) Schnappinger, T.; Kowalewski, M. Ab Initio Vibro-Polaritonic Spectra in Strongly Coupled Cavity-Molecule Systems. *J. Chem. Theory Comput.* **2023**, *19*, 9278–9289.

(2) Rokaj, V.; Welakuh, D. M.; Ruggenthaler, M.; Rubio, A. Light–matter interaction in the long-wavelength limit: no ground-state without dipole self-energy. *J. Phys. B At. Mol. Opt. Phys.* **2018**, *51*, 034005.

(3) Schäfer, C.; Ruggenthaler, M.; Rokaj, V.; Rubio, A. Relevance of the Quadratic Diamagnetic and Self-Polarization Terms in Cavity Quantum Electrodynamics. *ACS Photonics* **2020**, *7*, 975–990.

(4) Welakuh, D. M.; Rokaj, V.; Ruggenthaler, M.; Rubio, A. Non-perturbative mass renormalization effects in non-relativistic quantum electrodynamics. **2023**,

(5) Hopfield, J. J. Theory of the Contribution of Excitons to the Complex Dielectric Constant of Crystals. *Phys. Rev.* **1958**, *112*, 1555–1567.
